# Prognostic models in multiple sclerosis: progress and challenges in clinical integration

**DOI:** 10.1186/s42466-024-00338-z

**Published:** 2024-09-05

**Authors:** Joachim Havla, Kelly Reeve, Begum Irmak On, Ulrich Mansmann, Ulrike Held

**Affiliations:** 1 lnstitute of Clinical Neuroimmunology, LMU University Hospital, LMU Munich, Munich, Germany; 2 lnstitute of Clinical Neuroimmunology, Biomedical Center (BMC), Faculty of Medicine, LMU Munich, Munich, Germany; 3 Epidemiology, Biostatistics and Prevention Institute, University of Zürich, Zurich, Switzerland; 4 Institute for Medical Information Processing, Biometry and Epidemiology, Faculty of Medicine, Ludwig-Maximilians-Universität München, Munich, Germany

**Keywords:** Multiple sclerosis, Prognostic model, Treatment decision, Validation, Personalized medicine

## Abstract

As a chronic inflammatory disease of the central nervous system, multiple sclerosis (MS) is of great individual health and socio-economic significance. To date, there is no prognostic model that is used in routine clinical care to predict the very heterogeneous course of the disease. Despite several research groups working on different prognostic models using traditional statistics, machine learning and/or artificial intelligence approaches, the use of published models in clinical decision making is limited because of poor model performance, lack of transferability and/or lack of validated models. To provide a systematic overview, we conducted a “Cochrane review” that assessed 75 published prediction models using relevant checklists (CHARMS, PROBAST, TRIPOD). We have summarized the relevant points from this analysis here so that the use of prognostic models for therapy decisions in clinical routine can be successful in the future.

## Main text

Multiple sclerosis (MS) is a chronic inflammatory disease of the central nervous system with great individual health and socio-economic relevance [1]. To date, there is no prognostic model that is used in routine clinical care to predict the very heterogeneous course of the disease. Such a model could aid in personalized therapy selection under the assumption that those at highest risk benefit most from treatment. In this way, over-, under- and/or expensive mistreatment risk could be minimized. MS care providers are fortunate to have multiple therapeutic options with different modes of action, which differ significantly in terms of effectiveness, but also in terms of the risk-benefit balance. There is evidence that early, highly effective therapy improves the long-term outcome of MS progression, but at the price of a higher therapy-associated burden [2]. With the help of good prognostic model research, we move toward solutions to enable personalized therapy on an individual level. Although several research groups are working on different prognostic models using traditional statistics, machine learning, and/or artificial intelligence approaches [3], the use of published models in clinical decision making has been limited, due to poor model performance, lack of transportability and/or lack of validated models.

**Fig. 1:**
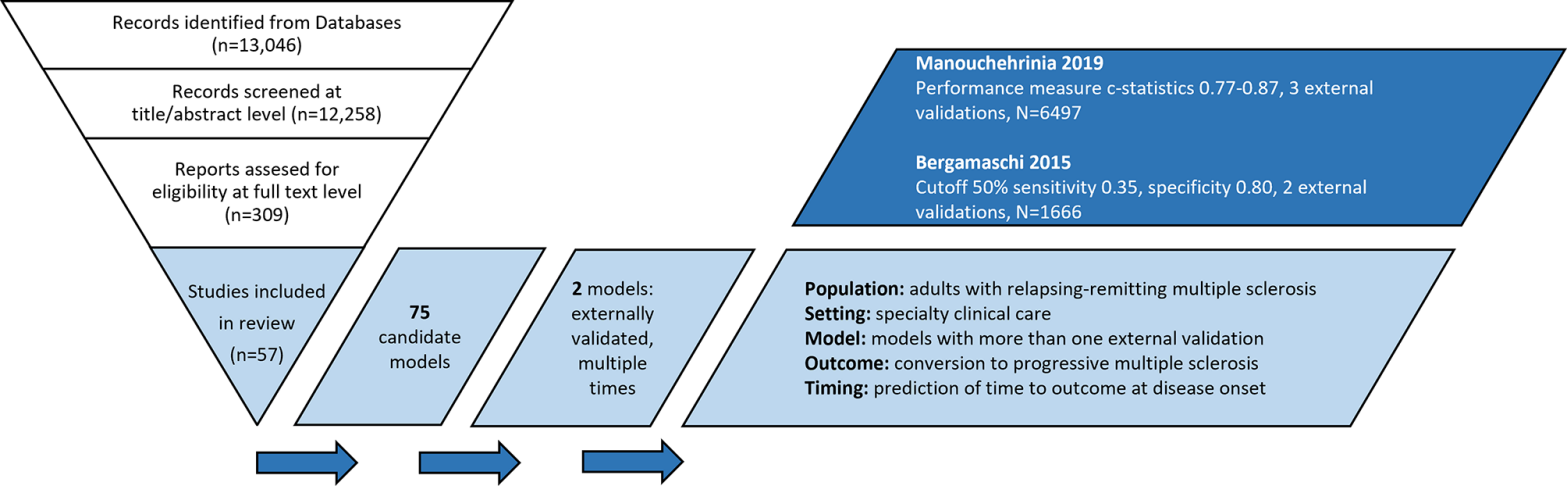
Summary of findings of [3]. Databases used for literature search: MEDLINE, EMBASE, Cochrane. 2 externally validated models: Manouchehrinia et al., 2019 [8] Bergamaschi et al., 2015 [9]

In order to provide a systematic overview we conducted a “Cochrane Review” assessing 75 prediction models published between January 1996 and July 2021 using prognostic modeling relevant checklists for data extraction (CHARMS), risk of bias assessment (PROBAST), and completeness of reporting (TRIPOD) [3]. Evidence on the performance of a prediction model can only be combined when there is at least three external validations outside the model development process, preferably led by independent researchers [4]. However, no published model met this gold standard [3]. Of these 75 candidate models, only 12 were externally validated at all and only two of these multiple times (Fig. 1). No external validations were performed by independent researchers. In addition, the comparability of the models was limited. The prognostic models used heterogeneous outcomes with different definitions, such as disease progression (41%), conversion to secondary progressive MS (28%), conversion to definitive MS diagnosis (18%) or occurrence of relapses (8%). On the other hand, the rapid development with an increase in treatment options, the availability of markers and the diversification of the diagnostic criteria severely limits the comparability of different cohorts and models. Some models show only limited applicability in non-specialized treatment settings. Furthermore, 52% of the prognostic models lacked clear reporting or instructions to enable their validation in other cohorts.

The following points would facilitate the translation of prognostic models for aiding treatment decisions in MS routine:

1. Prognostic model research should be undertaken by teams with expertise in MS and its treatment, in data collection process, in prediction algorithms, and all aware of the expectations of clinical prognostication and its reporting.
2. Independent external validation is a vital and ongoing process [5], especially in the dynamic MS domain with ever-changing disease definitions.
3. Model development publications clearly document the development and evaluation steps and guide the implementation following TRIPOD, including description of the intended time of model use and prediction horizon. The new TRIPOD + AI (Transparent Reporting of a multivariable prediction model for Individual Prognosis Or Diagnosis + Artificial Intelligence) provides harmonized guidelines for the reporting of prediction model studies, regardless of whether regression models or machine learning methods were used [6].

To give an example of how such a model development could be methodologically implemented, a multicenter prospective cohort study was planned and conducted solely for the purpose of external validation of a prognostic score, ensuring compliance with the methodological guidelines [7].

By applying above mentioned recommendations, future prognostic models could overcome limitations and contribute to personalized prognostication in people with MS [3].

## Data Availability

Not applicable.
